# Epidemiological study air disaster in Amsterdam (ESADA): study design

**DOI:** 10.1186/1471-2458-5-54

**Published:** 2005-05-30

**Authors:** Pauline Slottje, Anja C Huizink, Jos WR Twisk, Anke B Witteveen, Henk M van der Ploeg, Inge Bramsen, Nynke Smidt, Joost A Bijlsma, Lex M Bouter, Willem van Mechelen, Tjabe Smid

**Affiliations:** 1 Institute for Research in Extramural Medicine, VU University Medical Center, Van der Boechorststraat 7, 1081 BT Amsterdam, the Netherlands; 2 Department of Public and Occupational Health, VU University Medical Center, Amsterdam, the Netherlands; 3 Department of Child and Adolescent Psychiatry, Erasmus Medical Center, Rotterdam, the Netherlands; 4 Department of Clinical Epidemiology and Biostatistics, VU University Medical Center, Amsterdam, the Netherlands; 5 Department of Medical Psychology, VU University Medical Center, Amsterdam, the Netherlands; 6 KLM Health Services, Schiphol Airport, the Netherlands

## Abstract

**Background:**

In 1992, a cargo aircraft crashed into apartment buildings in Amsterdam, killing 43 victims and destroying 266 apartments. In the aftermath there were speculations about the cause of the crash, potential exposures to hazardous materials due to the disaster and the health consequences. Starting in 2000, the Epidemiological Study Air Disaster in Amsterdam (ESADA) aimed to assess the long-term health effects of occupational exposure to this disaster on professional assistance workers.

**Methods/Design:**

Epidemiological study among all the exposed professional fire-fighters and police officers who performed disaster-related task(s), and hangar workers who sorted the wreckage of the aircraft, as well as reference groups of their non-exposed colleagues who did not perform any disaster-related tasks. The study took place, on average, 8.5 years after the disaster. Questionnaires were used to assess details on occupational exposure to the disaster. Health measures comprised laboratory assessments in urine, blood and saliva, as well as self-reported current health measures, including health-related quality of life, and various physical and psychological symptoms.

**Discussion:**

In this paper we describe and discuss the design of the ESADA. The ESADA will provide additional scientific knowledge on the long-term health effects of technological disasters on professional workers.

## Background

In the early evening of October 4^th^, 1992, an El Al Boeing 747-F cargo aircraft lost two of its engines just after take off from Schiphol Airport and crashed into two apartment buildings in the Bijlmermeer, a densely populated suburb of Amsterdam (the Netherlands) [1]. The air disaster killed 43 people, and destroyed 266 apartments [1,2]. Fire-fighters and police officers were called to the scene to extinguish fires, to search and rescue people, to assist in the identification of human remains and personal belongings, to secure the surroundings and to clean-up the devastated area. Many of them were faced with bewildered residents and extensive destruction, and some witnessed dead or injured victims. Within a few days the wreckage of the aircraft was transported to a hangar at Schiphol Airport, where employees (i.e. 'hangar workers') sorted and inspected the wreckage.

In the extensive aftermath of the disaster, rumors and questions arose about the cause of the accident, the contents of the cargo, potential exposure to hazardous materials, and health consequences [2,3]. Every now and then the media highlighted stories of individual victims, as well as uncertainties about potential exposures during the disaster [4]. One of the major topics concerned exposure to depleted uranium from the aircraft's balance weights, particularly because some of the depleted uranium has never been recovered from the rubble [1]. However, the authors of a retrospective risk analysis "considered it improbable that the missing uranium had indeed led to the reported health complaints" [5]. Nonetheless, it appeared that a growing number of exposed workers and affected residents reported health complaints, which some of them attributed to the disaster [6]. Public and political unrest thus waxed and waned in the aftermath of the disaster [2,3]. Eventually, a parliamentary inquiry, that was held in 1998, recommended an epidemiological study on the health effects of the disaster [1].

About the same time, in 1998, the employers of professional fire-fighters and police officers in Amsterdam decided to start an independent assessment of the health status of professional workers involved in the disaster. The mayor of Amsterdam assigned their occupational health service, the KLM Health Services, to organize this assessment. The employer of the hangar workers at Schiphol Airport joined this initiative, as did government representatives of the affected inhabitants and volunteer workers. It was decided to offer a medical examination to all people involved in the air disaster, residents as well as assistance workers, and that an epidemiological study would be performed simultaneously by the Institute for Research in Extramural Medicine (EMGO Institute). In this paper we report on the design of the epidemiological study among professional assistance workers: the Epidemiological Study Air Disaster in Amsterdam (ESADA). Unfortunately, the epidemiological study among residents had to be cancelled, due to low response rates.

The ESADA is the first epidemiological study that has ever been conducted after a major technological disaster in the Netherlands. The aim of this study is to assess the long-term psychological and physical health effects of occupational exposure to the air disaster in Amsterdam on professional assistance workers, i.e. fire-fighters, police officers and hangar workers. Based on the scientific literature on the health effects of disasters, the main hypotheses of the ESADA concern unexplained physical symptoms [7-12], and post-traumatic stress symptoms and associated psychological symptoms [13-15]. Due to the fact that the ESADA originated partly from societal concerns, we considered it necessary to also include some additional outcomes that will answer questions for some of the affected people, which, in turn, might help to reassure them. These societal questions relate to depleted uranium, Mycoplasma species and carnitine levels in plasma. The first of these questions stems from the concerns about the depleted uranium from the aircraft's balance weights, described above. The other two questions are primarily based on an alleged resemblance between the symptoms of some of the people affected by the air disaster in Amsterdam and the symptoms of patients with chronic fatigue syndrome (CFS) and Gulf War (I) Syndrome (GWS). Although some authors may have suggested a link between these syndromes and Mycoplasma species [16-21] or carnitine deficiency [22-26], others have rejected the existence of such links [27-29].

In this paper we describe and discuss the design of the ESADA. More details on the (organization of the) ESADA can be found on its website [30].

## Methods / Design

### Design

The ESADA is designed as a historical cohort study, in which the health status of the professional fire-fighters, police officers and hangar workers who were occupationally exposed to the air disaster in Amsterdam is compared with the health status of reference groups of workers with the same jobs and employers at the time of the disaster, but who were not occupationally exposed to this disaster.

### Study population

The ESADA study population consisted of professional fire-fighters, police officers and hangar workers. Eligible subjects had to (1) sign informed consent; (2) have sufficient mastery of the Dutch language to fill in the questionnaires; and (3) belong to one of the following three occupational groups:

1) All professional fire-fighters who were, according to company records, employed in the Amsterdam fire department at the time of the disaster. Additional professional fire-fighters who started working in this fire department after the disaster were also invited to participate in the study, as almost the entire fire department had been exposed to the disaster.

2) All police officers (*i.e. *constables, warrant officers, sergeants and their supervisors) who were, according to company records, employed in the Amsterdam-Amstelland regional police force on the date of the disaster (October 4^th^, 1992), and were still employed there on the 1^st ^of January 2000.

3) All the hangar workers registered as working for one of the departments involved in the transport, security and sorting of the wreckage on the date of the disaster (October 4^th^, 1992), and who reported to have been involved in these activities; as well as a random sample, matched with their colleagues for age, sex, department and job title, who were also registered as working for these departments on 30^th ^November 1992, but who did not report to have been involved in any disaster-related activities.

### Procedures and data-collection

The study design was approved by the two independent Medical Ethics Committees of the medical facilities involved in this project: the VU University Medical Center (VUmc) and the 'Onze Lieve Vrouwe Gasthuis' (OLVG) in Amsterdam. Potential participants were initially informed about the study via announcements in staff magazines, after which they were approached via personal letters, and eventually by telephone. All participants signed informed consent and participated voluntarily. Data were collected at the Prinsengracht out-patient clinic of the OLVG from January 2000 to March 2002, i.e. on average 8.5 years after the disaster. In addition, data on about half of the hangar workers were collected at Schiphol Airport for logistic reasons. Trained medical research assistants checked that the questionnaires had been completed, measured body height and weight, drew blood samples, and assisted with the collection of urine and saliva samples. A team of administrative employees carried out the data-entry of the questionnaires. Data of each participant were entered twice by two of these employees independently, after which inconsistencies were reviewed and any mistakes rectified. All remaining problems in the interpretation of data, such as dubious handwriting, were consistently resolved by one of the authors (AH, PS or AW).

Blood, saliva and urine samples were dealt with according to standard procedures for collection, transportation, storage and laboratory analysis. Laboratory technicians could have been aware that the samples were from the ESADA, but they were blinded for exposure and health status. The laboratories were all certified according to accredited Dutch standards.

### Occupational exposure to the disaster

All participants were asked to fill in a questionnaire on occupational exposure to the air disaster. This questionnaire addressed several specific disaster-related tasks, and also the total time spent on these tasks and the location in which they were performed (e.g. on or near the disaster site, in the hangar where the wreckage was temporarily placed, or elsewhere). They were also asked to describe any other disaster-related task(s) that they had performed. Answers to the latter question were categorized (by PS and AW). The questionnaire also covered disaster-related psychosocial events in a number of items on personal experiences during the disaster (e.g. "were you in life-threatening danger?", "did you see the disaster scene during the first hours after the crash?", and "were any of your family members injured?").

These personal records of occupational exposure to the disaster were used to define 'exposed' workers, i.e. those who reported at least one disaster-related task, and 'non-exposed' workers, i.e. those who did not report any disaster-related tasks.

In addition to comparing exposed and non-exposed workers, we examined exposure-response relationships among exposed workers, in which level of exposure is characterized by the type of tasks and psychosocial events and the duration of exposure. As an additional dimension of level of exposure, we took into account the differences in potential psychotraumatic impact of exposure items, based on criterion A1 of the diagnostic criteria for Post Traumatic Stress Disorder (PTSD; American Psychiatric Association [APA]; Diagnostic and Statistical Manual of Mental Disorders-IV-Text Revision [DSM-IV-TR, 2000]) [31]. This criterion states that "the person has experienced, witnessed, or been confronted with an event or events that involve actual or threatened death or serious injury, or a threat to the physical integrity of oneself or others". Five experts on PTSD from different universities and psychiatric hospitals independently rated the likelihood of potentially psychotraumatic disaster-related tasks and events to meet this criterion on a 4-point Likert Scale ranging from 1 = 'very unlikely' to 4 = 'very likely'. Subsequently, we assumed that items with a mean item score of three or higher met the A1 criterion for PTSD (i.e. A1 tasks and events), as opposed to items with a lower mean score (i.e. non-A1 tasks and events). Table 1 lists the disaster-related tasks and the psychosocial events according to their potential psychotraumatic impact.

**Table 1:** Disaster-related tasks and psychosocial events according to their potential psychotraumatic impact

	A1* (traumatic)	Non-A1* (non-traumatic)
**Tasks**	1. Identification or recovery of victims from the rubble/transport or search for human remains 2. Rescue people	1. Fire-extinguishing 2. Clean up of destructed area 3. Transport of injured victims 4. Provide first aid/support injured victims or workers 5. Security tasks (surveillance, prevent burglary, keep disaster area free of bystanders) 6. Other tasks (e.g. traffic management) 7. Sort wreckage in hangar (at Schiphol Airport) 8. Other tasks in hangar in the presence of the wreckage 9. Transport of wreckage 10. Burning of contaminated soil remnants (from disaster site)

**Psychosocial events**	1. Having been in life-threatening danger during disaster 2. Personal injuries due to disaster 3. Witnessed dead or injured victims 4. Having been in or near one of the destroyed buildings at the time of the disaster 5. Immediate family members (partner, children) died / in life-threatening danger / injured due to the disaster 6. Other family members died due to the disaster	1. Saw the aircraft crash / saw or heard the aircraft when it crashed 2. Felt or heard the impact of the crash 3. Saw the fire 4. Saw the disaster site during the first hours after the crash/when the wreckage was still there 5. Other family members in life-threatening danger or injured due to the disaster 6. Friends or acquaintances died, injured or in life-threatening danger due to the disaster 7. Apartment of other family members, friends, or acquaintances damaged due to the disaster 8. Lived in the affected suburb of Amsterdam (Bijlmermeer) at the time of the disaster 9. Visited the hangar where the wreckage was kept

*A1 and non-A1 = items with a mean score of ≥ 3 or <3, respectively, on a 4 point Likert Scale indicating the likelihood for an item to meet criterion A1 for post-traumatic stress disorder (from 'very unlikely' [=1] to 'very likely' [=4]) (see Methods).

### Main health outcomes

#### Self-reported health measures

• *Post-traumatic stress symptoms*: (a) The Dutch 22-item Self-Rating Inventory for PTSD (SRIP) [32-34] and, among exposed subjects only, (b) The 15-item Dutch version of the Impact of Event Scale (IES), which addressed post-traumatic stress symptoms with explicit reference to the air disaster in Amsterdam [35-37].

• *General mental health*: (a) The 90-item Symptom Checklist (SCL-90) [38,39]; (b) The 20-item General Health Questionnaire (GHQ-12) [40].

• *Fatigue and associated symptoms*: The 20-item Checklist Individual Strength (CIS) [41,42].

• *Health-related quality of Life*: The MOS 36-item Short-Form Health Survey (SF-36) [43,44].

• *Chronic conditions*: One questionnaire assessed the current presence and history of the following chronic conditions, which are considered to have a significant impact on well-being: diabetes; stroke, brain hemorrhage or infarction; heart attack; other heart problems (such as heart failure, or angina pectoris); cancer; chronic osteoarthritis (wear) of the hip or knee joints; hypertension; asthma, chronic bronchitis or lung emphysema (Chronic Obstructive Pulmonary Disease [COPD]); serious or persistent intestinal disorders (longer than 3 months); chronic stomach disorders, stomach or duodenal ulcers; serious or persistent back complaints (including hernias); chronic inflammation of the joints (chronic rheumatism, rheumatoid arthritis). Workers with these chronic conditions were subsequently asked in what year the onset was, to determine whether this was before the disaster took place.

• *Physical symptoms*: Multiple questionnaires were used to assess the current presence of various physical symptoms, such as a number of respiratory, musculoskeletal, and skin symptoms.

• *Attribution of current problems to the air disaster in Amsterdam and its aftermath*. Another questionnaire assessed the extent to which exposed workers related any of their current physical, psychological or practical/financial problems to the air disaster and its aftermath. Those who attributed physical symptoms to the disaster and its aftermath were asked to specify these symptoms.

#### Laboratory outcomes

General laboratory tests [1]:

• *Hematological and blood chemical outcomes*: hemoglobin, leukocyte count, differential count, platelet count and mean corpuscular volume (Sysmex SE 9000, TOA medical electronics Co. ltd); potassium (Roche Modular ISE900, Roche Diagnostics); creatinine, alkaline phosphatase, gamma-glutamyl transferase, alanine aminotransferase, creatine kinase and C-reactive protein (Roche Modular P800, Roche Diagnostics); ferritin and thyroid stimulating hormone (Centauer, Bayer Diagnostics); β2-microglobuline (IMx Abbott).

• *Autoantibodies*: nuclear antigen antibodies, anti-double stranded DNA antibodies [46], Immunoglobulin (IgM) rheumatoid factor [47], antineutrophil cytoplasmic antibodies [48,49], and cardiolipin antibodies [50,51].

• *Urine outcomes*: creatinine (Hitachi 747, Roche Diagnostics GmbH, Mannheim, Germany); micro-albumin (Beckman Array 360 system); and β2-microglobuline (IMx Abbott); screening for protein, glucose, pH, blood and leukocytes (teststrip Boehringer Mannheim B.V.), followed by microscopic evaluation of the urinary sediment if indicated.

• *Saliva outcome*: cortisol concentration (Wizard 1470, Perkin Elmer).

Additional laboratory tests with respect to the societal questions:

• *Uranium 238*: concentration in urine (Inductively Coupled Plasma-Mass Spectrometry [ICP-MS] analyser, Finnigan Mat Element) and, at concentrations above 50 ng/l or above 50 ng/g creatinine, also the ratio of uranium 235/238 isotopes [52].

• *Total and free carnitine*: concentration in blood plasma (Mira Plus, Roche Diagnostics) [53,54].

• *DNA of any Mycoplasma species*: presence in peripheral blood mononuclear cells (DNA-isolation, Magna Pure, Roche Diagnostics; real time PCR, Taqman, Applied Biosystems); positive samples were subsequently evaluated for the presence of DNA of Mycoplasma fermentans [55,56].

### Self-reported socio-demographic characteristics

• *Age*: at time of assessment in years.

• *Sex: *male or female.

• *Ethnicity: *categorized into those who considered themselves as European (i.e. Dutch, British, Dutch/Irish, Dutch/Chinese, Dutch/Indonesian, Portuguese, Spanish, Dutch/ Spanish and European), and others (*e.g. *Moroccan, Turkish, Surinam).

• *Level of education*: highest level of education completed, categorized as: high (higher vocational education, university); medium (intermediate vocational education, higher general secondary education, or pre-university education); and low (no education, elementary school, lower vocational education, or lower general secondary education).

• *Current executive function*: yes (i.e. supervising one or more people) or no.

• *Level of physical activity*: the total number of hours spent each week on physical activities such as physical exercise, gardening and housekeeping, classified into high, medium and low according to the 33^rd ^and 66^th ^percentiles.

• *Alcohol consumption*: Usual and exceptional consumption of alcoholic beverages, classified into: none; light-moderate; and (extremely) excessive, i.e. consumption of (a) six or more glasses on 9–20 days a month and on 3–4 days in the last week, (b) four or more glasses on at least 21 days a month and on at least 5 days in the last week, and/or (c) more than six glasses a day, on a weekly basis.

• *Cigarette-smoking: *categorized as: never, former smoker, and current smoker.

• *Negative life events: *the number of reported negative life events, based on a questionnaire which specified 13 such events and also included two open-ended questions in which other events could be described. Subjects were asked to indicate whether any of these events happened to them before or after the disaster.

### Role of funding sources

The study was funded by the Dutch Ministry of Health, Welfare and Sports; the City of Amsterdam; the Amsterdam-Amstelland regional police force; and KLM Royal Dutch Airlines. The funding sources had no role in the collection, analysis, or interpretation of the data, or in the decision to submit a manuscript for publication.

## Discussion

In recent years there has been increasing scientific and societal interest in the health consequences of man-made, technological disasters, i.e. a collective stressful experience with a sudden onset due to technological failure. Technological disasters have had psychiatric consequences [13-15,57], such as PTSD, as well as medical consequences, in particular those of toxic exposures [58-61]. In addition to direct toxic health effects, the mere suspicion and fear of exposure to hazardous materials can also take its toll on the quality of physical, psychological and social well-being in the community [62-64].

Technological disasters strike unexpectedly and suddenly, which puts time-pressure on researchers to develop study protocols, gather exposure data, call in multidisciplinary experts, and obtain financial resources for immediate epidemiological research. Disaster researchers may also have to deal with complicated socio-political and legal aspects. In addition, they have to face a number of methodological problems. These difficulties include: (a) defining the entire potentially 'affected' population and appropriate reference groups; (b) contacting potential participants, particularly in the case of evacuation and hospitalization; low response rates; usually without data on non-respondents [65]; (c) collecting exposure data immediately after the event, which is actually also needed for long-term epidemiological studies.

Probably due to these difficulties, evidence from large-scale epidemiological studies that have been carried out after technological disasters is rather scarce [66,67]. Furthermore, before-after comparisons are rare and only possible by chance in ongoing research projects, due to the unexpected nature of technological disasters [68-70]. Most of the studies that have been conducted so far have relied on 'convenience samples', which were mainly composed of those who were directly affected, such as victims and residents; were based on non-epidemiological study designs; and used group-level or retrospective, self-reported exposure data, which can be affected by recall and reporting bias [71-74].

### ESADA approach

The purpose of the ESADA is to assess long-term health effects of occupational exposure to the air disaster in Amsterdam on professional assistance workers. In view of the above-mentioned difficulties in epidemiological research on disasters, the ESADA has some strong methodological points. With respect to the study population, we have been able to identify the complete cohort of exposed and non-exposed workers accurately, based on company records of employment at the time of the disaster.

Another strong point of the ESADA is that we included reference groups of colleagues, who had the same jobs and employers, but who were not occupationally exposed to the disaster. Hence, we are able to draw group-level conclusions on associations between health status and occupational exposure to the disaster.

With respect to exposure assessment, we were able to collect individual data on occupational exposure to the air disaster. Moreover, this consisted of multiple aspects of self-reported occupational exposure, including the duration and location of various disaster-related tasks and the experience of potentially stressful events during these tasks. Finally, we also included various assessments of long-term health, such as laboratory tests and self-reported symptoms and health-related quality of life, to obtain an integral evaluation of health status.

Notwithstanding these strong methodological qualities, some limitations of the ESADA design should also be mentioned. Firstly, although company records of employment were available, we still had to resolve a few difficulties regarding the definition of the study population. For the fire-fighters, this was due to the fact that almost the entire fire department of Amsterdam had been exposed to the disaster. Therefore, in order to achieve an adequate reference group, we decided to also include fire-fighters who joined this fire department after the disaster took place. With respect to the police officers, we were unable to trace those who had left the Amsterdam-Amstelland regional police force in the years after the disaster, due to administrative difficulties. Hence, it was necessary to restrict this group to those who were still working for this police force in 2000.

A second methodological issue concerns the self-report nature of occupational exposure status, and the average time-lag of 8.5 years between the disaster and the assessment. Due to administrative deficiencies in the historic registration of the exposure status, we used our detailed questionnaire data to define exposure status for all workers. Strictly speaking, the ESADA is therefore not a historic cohort study, but a cross-sectional one. The time-lag between the disaster and the exposure assessment may have led to recall bias, especially concerning certain details of exposure to the disaster, such as the duration of activities. However, it seems reasonable to assume that the workers did recollect whether or not they performed any as opposed to no disaster-related tasks, which was used to define occupational exposure status. It is therefore very unlikely that recall bias has resulted in (non-)differential misclassification of exposed and non-exposed workers. Nevertheless, recall bias should be kept in mind with respect to exposure-response relationships. We included multiple aspects of level of exposure, such as the duration and the potential psycho-traumatic character of disaster-related tasks, as it is unknown which aspect of occupational exposure to disasters is relevant for long-term health. However, we may still have missed other potentially relevant aspects, such as exposure to disaster-related media reports in the aftermath of the disaster [4,75].

Thirdly, we acknowledge the fact that, with the exception of the laboratory variables, we rely on self-reported health outcomes. However, most of the health questionnaires that we used have been validated and widely accepted, except for those used to assess the physical symptoms. Differential misclassification in self-report health measures could occur if exposed workers are more likely than non-exposed workers to interpret and report bodily sensations as symptoms. On the other hand, hypervigilance and hypochondria themselves could well be adverse health effects of (toxicological) disasters [76,77].

In conclusion, to increase our knowledge of potential health consequences of (technological) disasters, it is important to be prepared for epidemiological disaster research. Incorporating basic multidisciplinary, epidemiological research protocols into disaster management plans will stimulate scientifically sound research on the health effects of disasters. The ESADA will provide additional scientific knowledge on the long-term health effects of technological disasters on professional workers.

## Competing interests

The author(s) declare that they have no competing interests.

## Authors' contributions

All authors participated in the multidisciplinary ESADA project team of the EMGO Institute, provided comments on the draft versions and approved the final manuscript. PS drafted the manuscript and performed statistical analyses. AH coordinated the acquisition of data and supervised the first part of the ESADA study. JT contributed to the design and supervised the statistical analyses. AW coordinated the rating of the potential psychotraumatic impact of exposure items and performed statistical analyses. HP and IB participated in the conception and design of the ESADA with respect to psychological outcomes, and IB rated the potential psychotraumatic impact of exposure items. NS supervised the second part of the ESADA. As Medical Director JB coordinated the data-collection. LB contributed to the design of the ESADA. WM contributed to the design and is Vice-President of the ESADA project team. TS conceived the study, and participated in its design and coordination as President of the ESADA project team.

## Pre-publication history

The pre-publication history for this paper can be accessed here:


